# Postoperative clinicopathological factors affecting cervical adenocarcinoma: Stages I–IIB

**DOI:** 10.1097/MD.0000000000009323

**Published:** 2018-01-12

**Authors:** Jiansong Zhou, Yuanyuan Chen, Xiaoxian Xu, Dingding Yan, Hanmei Lou

**Affiliations:** a Key Laboratory of Radiation Oncology of Zhejiang Province; b Department of Gynecologic Radiation Oncology, Zhejiang Cancer Hospital; c Department of Radiation Oncology, Hangzhou Cancer Hospital, Hangzhou, Zhejiang, P.R. China.

**Keywords:** adenocarcinoma, cervical cancer, pelvic node metastasis, prognostic factors

## Abstract

Currently, cervical adenocarcinoma (ADC) receives the same standard treatment as squamous cell carcinoma, but this treatment regimen is not wholly suited for ADC. The present study was conducted to assess the prognostic role of postoperative clinicopathological factors in patients with stage I–IIB cervical ADC.

The study examined 312 patients with stage I–IIB cervical ADC who underwent radical hysterectomy, including pelvic lymphadenectomy, at our institutions between October 2006 and September 2014. Overall survival (OS) and relapse-free survival (RFS) was analyzed by the Kaplan–Meier method. Sites of recurrence were classified as local and distant locations.

The 5-year OS and RFS rates were 88.2% and 83.8%, respectively. The 5-year OS rates for patients with International Federation of Gynecology and Obstetrics (FIGO) stage IA, IB, IIA, and IIB were 100.0%, 90.7%, 82.8%, and 55.6%, respectively. The Cox model identified number of positive pelvic nodes and age at surgery as independent prognostic factors for survival, and number of positive pelvic nodes and postoperative tumor diameter (≥4 cm) as independent prognostic factors for relapse. Cancer recurrence developed in 35 women. The top three recurrence sites were pelvis, vaginal stump, and lung.

A more aggressive therapeutic strategy different from current practice in cervical cancer is urgently required for cervical ADC. As a new prognostic factor, postoperative tumor diameter should receive special attention in ADC treatment.

## 1 Introduction

Invasive cervical cancer (ICC) ranks third as the most common malignancy and fourth as the cause of cancer-related deaths among women worldwide.^[1]^ Despite population-based screening and development of advanced medical treatments, the morbidity of ICC is still common in developing countries like China, yielding 132,300 new cases each year.^[2]^

Currently, adenocarcinoma (ADC) receives the same standard treatments as squamous cell carcinoma (SCC): radical hysterectomy, radical hysterectomy followed by adjuvant radiotherapy (RT) or primary RT for early-stage carcinoma. Concurrent chemoradiotherapy (CCRT), which is recommended in cases of locally advanced cancer and for patients with FIGO early-stage disease, has been widely accepted^[3]^ and produces equivalent results. However, disparate prognoses have been observed in both SCC and ADC patients with the same stage based on FIGO guidelines.^[4,5]^ Moreover, an upward trending incidence of ADC has been reported in many countries.^[6,7]^ This upward trend is particularly evident among women under age 40.^[8,9]^ The proportion of ADC has doubled over the past decade and accounts for approximately 25% of all cases of cervical cancer.^[10]^ The poorer prognosis of ADC patients compared to those with SCC raises the question of whether the current standard treatment for patients with SCC is suitable for those with ADC.^[11]^ Thus, it is of great importance to determine the prognostic factors involved in ADC so as to establish a framework for new therapeutic strategies.

In the literature, the prognostic significance of some clinicopathological factors still remains controversial.^[12,13]^ The aim of the present retrospective study was to clarify the clinicopathological factors for predicting the prognosis for ADC.

## 2 Materials and methods

All consecutive patients diagnosed with the FIGO stage I–IIB invasive ADC of the uterine cervix and treated at the Zhejiang Cancer Hospital, Zhejiang Province, China between October 2006 and September 2014 were eligible for this study. Briefly, all of the patients underwent primary surgery consisting of radical hysterectomy with pelvic lymphadenectomy. Patients with postoperative pathological risk factors, such as bulky tumor size, deep stromal invasion (DSI ≥ 1/2), lymph-vascular space invasion (LVSI), lymphatic metastases and parametrial and surgical margin involvement were advised to receive adjuvant RT or CCRT. Radiotherapy (RT) was performed with a total of 45 to 50 Gy of external beam RT, with or without platinum-based chemotherapy. Clinical data were extracted from the institutions’ electronic databases. Follow-up data were obtained through correspondence and/or outpatient department visits. The Medical Ethics Committee of Zhejiang Cancer Hospital approved the study.

### 2.1 Statistical analysis

Statistical analyses were performed with the SPSS 16.0 software package (IBM, Armonk, NY). *P* values < .05 were considered statistically significant. Summary statistics are presented as frequencies and percentages. Overall survival (OS) and relapse-free survival (RFS) were obtained by the Kaplan–Meier method for different groups. The log-rank test was used to compare survival curves. Variables that showed a significant association with survival were included in multivariate analysis based on the Cox proportional–hazard model. Sites of recurrence were classified as local if detected in the pelvis or vagina, and distant if detected in extrapelvic locations.

## 3 Results

### 3.1 Patient characteristics

A total of 313 patients were identified, including 9 patients with stage IA, 218 patients with stage IB, 74 patients with stage IIA and 12 patients with stage IIB ADC. Patients’ characteristics and treatment modalities are summarized in Table 1. Median age of patients with ADC was 46 years (range: 19–73). Following surgery, 157 patients received adjuvant therapy. The median follow-up period was 56.46 months, ranging from 1 to 60 months. Patients’ recurrence status is shown in Table 2. One patient with stage IIA died from a traffic accident 5 months after therapy and was removed from our data. Three cases were lost during follow-up. RT alone and CCRT were carried out in 37 and 120 cases, respectively.

**Table 1:**
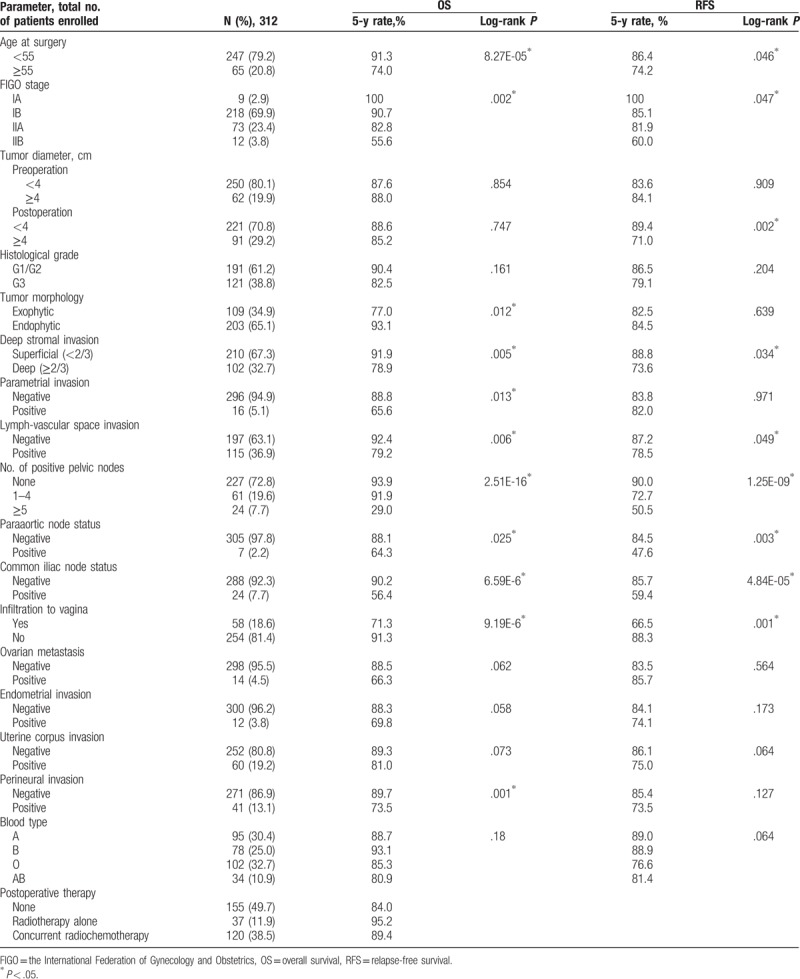
Patients’ characteristics, relapse-free survival, and overall survival in cervical adenocarcinoma.

**Table 2:**
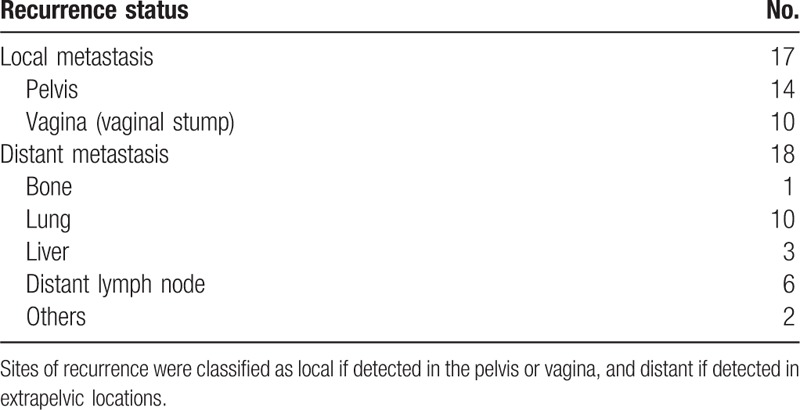
Recurrence status of cervical adenocarcinoma with radical hysterectomy and pelvic lymphadenectomy.

### 3.2 Survival

The OS and the RFS of the patients with ADC were assessed by log-rank test in 17 clinicopathological subgroups (Table 1). The 5-year OS and RFS rates were 88.2% ± 2.3% and 83.8% ± 2.6%, respectively. The 5-year OS rates for patients with FIGO stage IA, IB, IIA, and IIB were 100.0%, 90.7%, 82.8%, and 55.6%, respectively (*P* = .002). Univariate analysis identified as significant factors age at surgery (*P* = 8.27E-05), FIGO stage (*P* = .002), tumor morphology (*P* = .012), DSI (*P* = .005), parametrial invasion (*P* = .013), LVSI (*P* = .013), paraaortic node status (*P* = .025), common iliac node status (*P* = 6.59E-6), number of positive pelvic nodes (*P* = 2.51E-16), infiltration to vagina (*P* = 9.19E-6), and perineural invasion (PNI; *P* = .001). However, there were no significant differences in OS for pre/postoperation tumor diameter, histological grade, ovarian metastasis, endometrial invasion, uterine corpus invasion, and blood types. For these factors, multivariate analysis testing was performed to examine differences in survival among statistically distinct subgroups. The Cox model identified number of positive pelvic nodes (95% CI: 1.995–7.278, *P* = 5.09E-5) and age at surgery (95% CI: 1.574–7.502, *P* = .002) as independent prognostic factors for OS.

Similarly, RFS was also assessed in the subgroups using the same parameters as OS. Univariate analysis identified age at surgery (*P* = .046), FIGO stage (*P* = .047), postoperation tumor diameter (4 cm; *P* = .002), DSI (*P* = .034), LVSI (*P* = .049), number of positive pelvic nodes (*P* = 1.25E-09), paraaortic node status (*P* = .003), common iliac node status (*P* = 4.84E-05), and infiltration to vagina (*P* = .001) as independent prognostic factors for OS. The Cox model showed that postoperation tumor diameter (4 cm) (95% CI: 1.011–3.727, *P* = .046) and number of positive nodes (95% CI: 1.687–5.334, *P* = 1.83E-4) were independent prognostic factors for RFS (Table 3).

**Table 3:**
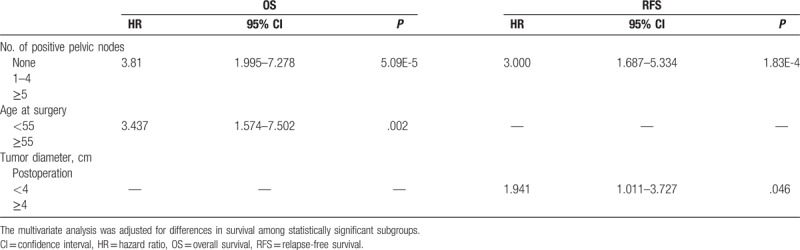
Multivariate analysis of prognostic factors for OS and RFS in 312 patients with adenocarcinoma.

### 3.3 Lymphatic metastasis pattern and recurrence sites

Of the 312 patients with ADC, the positive rate of pelvic lymph node metastasis after the initial surgery was 27.3% (85 of 312); among them, 24 patients had common iliac node metastasis. Furthermore, a positive rate of paraaortic node metastasis was observed in 7 (2.2%) patients.

At 5 years, 35 of 312 women (11.2%) suffered a cancer recurrence. Of these, 17 cases had local metastasis (angina or pelvis) and 18 cases had distant metastasis. The most frequent distant recurrence site was lung (10 patients, 55.6%), followed by the distant lymph node (6 patients, 33.3%), liver (16.7%), bone, and others (Table 2).

## 4 Discussion

Cervical cancer is still a leading cause of cancer-related death in women worldwide. For decades, ICC subtypes have received the same standard treatments.^[4,5]^ However, increasing evidence shows that ADC displays different HPV types, patterns of spread, prognosis and recurrence from SCC, as well as greater radioresistance and a higher rate of lymph node metastasis compared to SCC.^[14–17]^ ADC is associated with poorer prognosis. Furthermore, the proportion of ADC has doubled over the past decades, both in absolute and relative terms, compared with SCC.^[6]^ This trend may be due to the well-organized screening and early therapeutic intervention leading to decreased morbidity and mortality in SCC. Another reason is that preinvasive ADC (in situ and endophytic ADC) may often be missed through routine screening, especially when located in the endocervical canal.^[14,18]^ As a result, ADC tends to be diagnosed at a more advanced stage and is prone to a worse outcome than SCC.^[19]^ Subset analyses of several studies suggest higher recurrence rates after radiation in ADC compared to SCC.^[20]^ Hence, a more aggressive therapeutic strategy different from the currently adopted practice for SCC is urgently required.

The treatment of women with ADC is challenging, and the accurate prediction of cancer control after definitive treatment for ADC is also of great importance for patient counseling, follow-up, and treatment planning.^[21]^ Thus far, the prognostic significance of several clinicopathological factors remains uncertain. The present retrospective study examined 17 available parameters using univariate as well as multivariate analysis to illustrate their prognostic role in ADC. In addition, lymphatic metastasis pattern and recurrence site data were also collected.

By consulting the literature, a series of factors were identified as affecting survival in cervical ADC, including age, FIGO stage, tumor size (preoperation), DSI, number of positive nodes, LVSI, parametrial invasion, infiltration to vagina, ovarian metastases, histological grade, HPV genotype 18 and PNI.^[22–26]^ Kasamatsu et al^[27]^ identified tumor size and node metastasis as independent prognostic factors for survival, and infiltration to vagina and node metastasis as independent prognostic factors for relapse. Xia et al^[28]^ summarized parametrial invasion and pelvic node metastasis as independent prognostic factors for both survival and disease-free survival. In our study, FIGO stage was the most important prognostic parameter for ADC, with 5-year OS rates for patients with FIGO stage IA, IB, IIA, and IIB of 100.0%, 90.7%, 82.8%, and 55.6%, respectively. LVSI has also been shown to be negatively correlated with the 5-year survival rate in patients with ADC. Recent reports have indicated that LVSI could predict an increased risk (up to 32%) of positive lymph nodes.^[29]^ Parametrial involvement has also been reported to significantly influence outcome of patients.^[30]^ Here, we revealed that parametrial involvement influenced OS but not RFS. PNI is defined as tumor cell infiltration into a nerve or around nerve tissue, and PNI is considered to be evidence of metastasis through the nerve.^[31]^ The present study found that PNI (*P* = .001) was a predictive factor for OS. Endophytic ADC is an easily missed diagnosis and is associated with poorer prognosis. In the literature, the effect of tumor histology on ICC outcomes is uncertain and conflicting results have been reported. Other factors in our study, such as DSI and infiltration to vagina, also influenced survival in ADC.

In previous studies, tumor size has been shown to be an independent risk factor for survival, with bulky tumor size defined in most studies as a maximum diameter over 4 cm which was linked to the incidence of lymph node involvement^[32]^ and was found to be associated with poor outcome.^[29,33]^ The most prominent results in the present study was that postoperation (but not preoperation) tumor diameter at 2 cm (*P* = .0173), 3 cm (*P* = 9.78E-04), 4 cm (*P* = .002), and 5 cm (*P* = .023) cm was an independent prognostic factor for RFS, rather than OS. This result is unique among previous studies and illustrates the importance of the postoperation tumor diameter as a predictor for ADC. As an available and objective factor, postoperation tumor diameter is a new prognostic factor for ADC, and bulk postoperation tumor diameter can be expected to help in deciding upon the need for adjuvant therapy. Although several studies have suggested that younger age is an unfavorable prognostic factor, especially in more advanced stages, and survival analysis revealed that younger patients showed impaired survival,^[21]^ we regarded age as a controversial prognostic factor, which in spite of age at surgery (50 years (*P* = .004), 55 years (*P* = .002) and 60 years (*P* = .001)), was an independent prognostic factor for OS. Positive node is also an acknowledged independent adverse prognostic factor for survival and relapse of patients with FIGO stage I–IIB disease in ADC.^[27]^ Our data are consistent with previous studies showing that both 5-year OS (93.9% vs 29.0%) and 5-year RFS (90.0% vs 50.5%) were significantly better in patients without pelvic lymphatic metastasis. These results highlight the prognostic value of lymphatic metastasis (both paraaortic node and pelvic node) in ADC, which stands in sharp contrast to the status of ovarian metastasis, endometrial invasion, and uterine corpus invasion.

Most cervical cancer patients die of recurrent or metastatic diseases, especially distant metastasis. In our study, 35 of 312 women suffered cancer recurrence in the 5-year observation window. The distant recurrence sites were the usual sites; in order, lung, distant lymph nodes, liver, bone, and others. Adjuvant chemotherapy may contribute to eradicate subclinical distant metastases.^[34,35]^

There are several limitations in our study. First, we only enrolled women with primary cervical ADC treated in Zhejiang Cancer Hospital between October 2006 and September 2014. Second, our study was limited to the examination of only 17 clinicopathological subgroups. Other factors which may be associated with the prognosis for ADC, including high-risk HPV DNA load, age at first intercourse, endogenous and exogenous hormonal factors, obesity and infection with sexually transmitted infectious agents, were not included or discussed in our study.

In conclusion, our data showed that number of positive pelvic nodes and age at surgery were independent prognostic factors for OS, and postoperation tumor diameter (≥4 cm) and number of positive nodes were independent prognostic factors for RFS. We should also pay special attention to lymphatic metastasis and postoperation tumor diameter in ADC treatment. These data are important in increasing our understanding of the prognostic role of clinicopathological factors in ADC. Additionally, these data may provide information for further design of new therapeutic strategies which may be more suitable for ADC patients.

## Acknowledgments

The authors are very grateful to Jin Wang who helped with this study and is from Department of Gynecologic Radiation Oncology, Zhejiang Cancer Hospital.
